# Compound Risk of Air Pollution and Heat Days and the Influence of Wildfire by SES across California, 2018–2020: Implications for Environmental Justice in the Context of Climate Change

**DOI:** 10.3390/cli10100145

**Published:** 2022-10-01

**Authors:** Shahir Masri, Yufang Jin, Jun Wu

**Affiliations:** 1 Department of Environmental and Occupational Health, Program in Public Health, University of California, Irvine, CA 92697, USA; 2 Department of Land, Air, and Water Resources, University of California, Davis, CA 95616, USA

**Keywords:** climate change, heatwaves, air pollution, purpleair, PM_2.5_, compound risk

## Abstract

Major wildfires and heatwaves have begun to increase in frequency throughout much of the United States, particularly in western states such as California, causing increased risk to public health. Air pollution is exacerbated by both wildfires and warmer temperatures, thus adding to such risk. With climate change and the continued increase in global average temperatures, the frequency of major wildfires, heat days, and unhealthy air pollution episodes is projected to increase, resulting in the potential for compounding risks. Risks will likely vary by region and may disproportionately impact low-income communities and communities of color. In this study, we processed daily particulate matter (PM) data from over 18,000 low-cost PurpleAir sensors, along with gridMET daily maximum temperature data and government-compiled wildfire perimeter data from 2018–2020 in order to examine the occurrence of compound risk (CR) days (characterized by high temperature and high PM_2.5_) at the census tract level in California, and to understand how such days have been impacted by the occurrence of wildfires. Using American Community Survey data, we also examined the extent to which CR days were correlated with household income, race/ethnicity, education, and other socioeconomic factors at the census tract level. Results showed census tracts with a higher frequency of CR days to have statistically higher rates of poverty and unemployment, along with high proportions of child residents and households without computers. The frequency of CR days and elevated daily PM_2.5_ concentrations appeared to be strongly related to the occurrence of nearby wildfires, with over 20% of days with sensor-measured average PM_2.5_ > 35 μg/m^3^ showing a wildfire within a 100 km radius and over two-thirds of estimated CR days falling on such days with a nearby wildfire. Findings from this study are important to policymakers and government agencies who preside over the allocation of state resources as well as organizations seeking to empower residents and establish climate resilient communities.

## 1. Introduction

In a recent report, the Intergovernmental Panel on Climate Change (IPCC) stated that “it is unequivocal that human influence has warmed the atmosphere, ocean and land [1].” In the absence of major near-term reductions in CO_2_ and other human-related greenhouse gas emissions, the IPCC estimates that the global average surface temperature will continue to rise until at least the mid-century under all emissions scenarios, and that global warming of 1.5 °C and 2 °C will be exceeded during the 21st century. With increasing temperatures, the likelihood of experiencing days characterized by extreme heat, wildfire, and elevated air pollution are similarly expected to rise, thus posing a major threat to public health [1].

As it relates to temperature, extensive epidemiological evidence has documented a positive association between increased temperature and a range of adverse health outcomes including preterm birth, heat stroke, and all-cause mortality [2–7]. The adverse health impacts of high temperatures are reported to occur during both individual high-temperature days as well as during sustained periods of high temperature known as heatwaves [8–10].

In California, the National Oceanic and Atmospheric Administration (NOAA) has reported an increase in annual average temperature of approximately 1.5 °C (2.7 °F) from 1895 to 2021 (see Figure S1), equating to a 0.1 °C (0.2 °F) increase in average temperature per decade [11]. This temperature rise is about 35% greater than that of the nation as a whole (+1.1 °C) over the same period. Hulley et al. (2020) reported a three-fold increase in heatwave frequency over the past two decades in southern California, likely to increase by 42%, along with a 26% increase in duration, during future severe drought conditions [12]. Importantly, the authors also noted that periods of extreme heat have been extending later in the year, coinciding with the peak wildfire season, thus exposing populated regions to the simultaneous risks of extreme heat, wildfire, and pollution [12].

As temperatures have increased in recent decades, major wildfires have also occurred with increased frequency, as demonstrated by the nearly five-fold increase in the number of so-called “billion dollar” wildfire disasters that have occurred nationally between the 1980–1999 period compared to 2000–2019 [13]. Such fires have totaled to over $75 billion (USD) in damages in the U.S. alone since 2000 [14], a number which does not take into account the health-related costs from smoke exposure (also estimated in the billions of dollars) [15]. Although wildfire frequency cannot be ascribed exclusively to climate-related factors, the California Department of Forestry and Fire Protection (CAL FIRE), the state’s primary fire agency, indeed highlights climate change as a factor contributing to the exacerbation of wildfires [16]. Due in part to a drier climate, the western U.S. tends to experience the majority of the nation’s wildfire burning, in terms of area [17], with one study showing a four-fold increase in the occurrence of major wildfires and a 78-day increase in the length of the wildfire season across the region in recent decades [17].

On a state-by-state basis, California has been among the hardest hit by wildfires, with over half of the state’s top 20 largest wildfires occurring in just the last decade [18]. Of its six largest wildfires, five occurred in just two months in 2020 [18], including the record-breaking August Complex Fire that burned over 4000 km^2^ (1 M acres), more than doubling the area burned by California’s former 2018 record holder (Mendocino Complex Fire) [18]. As drought conditions and extreme winds become more frequent and vegetation more dry, the risk of major wildfires is projected to grow [16,19]. In our prior work examining wildfire frequency and burn area over a 21-year period in California, we documented a 73% increase in area burned across the state from 2011–2020 compared to 2000–2009, corresponding to an over 260 km^2^/year (64,000 acre/year) increase in wildfire-burned area over the study period [20].

While wildfire episodes come with direct adverse health effects including fire-related mortality and mental stress [21–25], such episodes are also characterized by indirect effects such as smoke-related air pollution exposure. Previous studies focusing on wildfire-related air pollution have documented 2- to 4-fold increases in concentrations of particulate matter with an aerodynamic diameter <2.5 μm (PM_2.5_), a form of air pollution widely associated with respiratory disease, cardiovascular disease as well as all-cause mortality and hospital admissions [22,26,27]. In some cases, PM_2.5_ levels rise over 10-times (>230 μg/m^3^) higher than background levels during wildfire episodes [28,29].

Mechanisms by which heat exacerbates respiratory diseases include increased inflammation (both systemic and pulmonary inflammation) resulting from thermoregulation as well as impairment of breathing patterns, cell damage, and blood clotting [30]. Air pollutants such as PM_2.5_ similarly produce inflammation through increased oxidative stress, leading to increased permeability of the airway epithelium, increased airway hyperresponsiveness, and decreased lung function [30]. In a recent systematic review of 56 different studies, Anenberg et al. (2020) affirmed strong evidence of synergistic health effects between heat and PM_2.5_ exposure, highlighting the threatening implications under climate warming scenarios [30].

As it relates to air pollution, traditional government-operated monitoring stations have historically suffered from uneven and sparse distribution, which has limited their ability to measure air pollution variability at a local scale [31]. In recent years, however, technological innovation has led to the development and deployment of thousands of low-cost air pollution sensors around the world, which have enabled scientists to characterize air quality at a high spatial and temporal resolution [32–34]. In 2017, for instance, the PurpleAir company began to deploy low-cost air quality sensors which now provide real-time PM_2.5_ data throughout the U.S. and abroad. A recent study of air pollution data in California confirmed such sensors to offer an improved representation of PM_2.5_ spatially when compared to regulatory monitoring stations [35]. Additionally, given their high density and real-time measurements, low-cost sensors allow for more accurate Air Quality Index reporting during wildfire and other extreme air pollution events [36]. Additionally, their low cost, mobility and simple maintenance allows these sensors to be readily owned and operated by governments, organizations, and individuals alike, which has helped this technology to expand regionally, as well as enabled citizens to actively participate in air pollution monitoring and awareness growth.

Given the relationship between heat waves, wildfires, and air pollution, these health threats are all likely to increase under future global warming [1]. What is more, although wildfire smoke is expected to increase, air pollution is also projected to worsen due to the relationship between temperature and the formation of photochemical air pollutants such as ozone [37]. The combined deleterious role of high temperature and high PM_2.5_ (exacerbated by the increasing frequency of major wildfires), especially in socioeconomically disadvantaged communities, presents a demonstrable potential for synergistic health outcomes with accompanying public health significance [30]. This “compound risk” is particularly plausible and critical to understand as dramatic heat extremes begin to coincide with the wildfire season in California [12]. What is more, the regional distribution of compound risk and its potential relationship with socioeconomic factors is important to examine so as to ensure adequate resource availability and preparedness for vulnerable communities.

In our prior work, we found that socioeconomically disadvantaged communities tended to sustain the greatest wildfire impact at the census tract level [20], underscoring the potential for greater risk in terms of their ability to evacuate and/or recover from a major wildfire [23,38]. Literature has also documented disproportionate exposure to air pollution and other environmental hazards among such communities [39–45]. Regarding heatwaves, a recent study by Xu et al. (2020) showed socioeconomic factors to be associated with temperature-related hospital admissions in Brazil, due to differences in adaptive capacity, work environment, and/or underlying risk factors [46]. Similar associations have been found in the United States [47]. Importantly, we are aware of no studies to date that identify high-risk communities as determined by the compound occurrences of extreme heat days and air pollution, nor their spatial relationship with socioeconomic factors at the community level.

In this study, we examined the frequency of days characterized by both high temperature and high PM_2.5_ concentrations (termed “compound risk days”) at the census tract level across the entire state of California during the three-year period from 2018 to 2020 in order to identify the most highly impacted communities and to describe them in terms of their socioeconomic characteristics. Specifically, our research questions were: (1) Which census tracts experienced the greatest number of high-temperature days and, separately, the greatest number of high-PM_2.5_ days, in California from 2018 to 2020? (2) How do census tracts experiencing the greatest number of high-temperature days overlap with those experiencing the most high-PM_2.5_ days? (3) What proportion of days with these compounding impacts are influenced by the occurrence of a nearby wildfire? and (4) Is the spatial distribution of census tracts experiencing CR days correlated with socioeconomic factors?

## 2. Data and Methods

### 2.1. Demographic Data

We used the most recently available (2010) Census data to obtain population counts for all census tracts (n = 8057) across the state of California. The American Community Survey (ACS), conducted every year, was also used to obtain information about household income, race/ethnicity, education, insurance coverage, age and other socioeconomic characteristics at the census tract level. For the ACS, five-year averaged data from 2018 was used since averages provide a more stable representation of community-level factors, and because 2018 was the most recent year for which five-year average data was available and since wildfire burned area was greatest in the more recent, as opposed to earlier, time period of the study. We did not utilize ACS data across multiple years of the study period so as to eliminate potential changes in social and economic characteristics that may fluctuate slightly from year to year, particularly after a major wildfire, as well as characteristics such as population size, home values, and income, which tend to exhibit positive trends over time irrespective of wildfire activity.

### 2.2. Temperature Data

We used gridMET daily maximum temperature data (4 km spatial resolution) produced by the Climatology Lab of the University of California, Merced [48]. The so-called gridMET data combines spatial attributes of gridded climate data from the Parameter-Elevation Regressions on Independent Slopes Model (PRISM). PRISM uses key inputs from approximately 10,000 weather stations as well as a weighted regression scheme to account for complex climate regimes. It also uses desirable temporal attributes along with additional variables from regional reanalysis of the North American Land Data Assimilation System Phase 2 data using climatically aided interpolation [49]. In this study, daily maximum temperature data was averaged by census tract to enable a regional comparison and demographic analysis.

### 2.3. PM_2.5_ Data

We downloaded 10-minute interval PurpleAir PM_2.5_ data for all sensor devices (n = 18,049) and all days spanning 2018 through 2020 using the ThingSpeak’s API provided by the PurpleAir company [50]. We did not use any data prior to 2018 since this was the first full year during which the PurpleAir network was in operation. The latest model of PurpleAir sensor model (PA-II-SD) contains two PMS5003 instruments, which estimate particle mass concentrations by measuring the amount of light scattered at ~680 nm [51]. Each sensor also recorded temperature and relative humidity, along with geographic coordinates, uptime (time during which a sensor is in consecutive operation from last boot time) and total sensor operating time (duration between measurement time and installation time). In order to reduce the impacts of potential sensor malfunction, intrasensor bias, and other environmental and operational parameter impact, all PurpleAir measurements underwent a two-step pre-processing procedure that included both quality control and calibration. The quality control procedure included the following four steps, which are similar to those described elsewhere [52,53].

1. Remove malfunctioning sensor data based on a low frequency of change (5-day moving standard deviation of zero) in their reported measurements over time.
2. Set PM_2.5_ outliers that exceed the sensor’s effective measurement range (daily values > 500 μg/m^3^) to 500 μg/m^3^.
3. Identify periods of prolonged interruption or data loss due to power outages or data communication loss using a 75% completeness criterion (≥108 10 min measurements in a day).
4. Examine the correlation from dual-channel readings for each sensor within a given month of operation based on calculated statistical anomality detection indicators as the coefficient of determination R^2^ > 0.8 and mean absolute error < 5.

Approximately 3.1 million daily PM_2.5_ observations were available during the three-year study period. This data set was reduced by 0.8%, 0%, 3.0%, and 2.9%, respectively, after following the steps described sequential above. Regressing U.S. Environmental Protection Agency’s (EPA) air quality system (AQS) data against post-processed PurpleAir sensor data yielded an R^2^ of 0.78 and slope of 1.0, which was higher than that of pre-processed data (R^2^ = 0.62). These results agree with previous work that has demonstrated stronger statistical agreement with AQS data based on the quality-controlled sensor data [35].

To ensure the consistency with the standard Ambient Air Quality Standard (AAQS) measurements, we calibrated a linear multivariate regression model to estimate outdoor PM_2.5_ concentrations from the PurrpleAir measurements (using measurements from co-located AQS regulatory stations and PurpleAir sensors). Co-location was defined as an AQS station falling within a 500 m radius of a PurpleAir sensor (determined using ArcGIS software). In total, 55 co-located sites were obtained, resulting in 24,994 matched daily measurements. Similar to other calibration approaches using co-located PurpleAir and AQS measurements [52,53], covariates including PA-measured temperature and relative humidity (RH) were incorporated into the calibration model. Additionally, the total sensor operating time and uptime were used to adjust for the potential effect of sensor aging and operational stability, respectively. Only uptime was found to be statistically significant (*p* < 0.05) and therefore retained for calibration.

### 2.4. Wildfire Data

**Figure 1.**
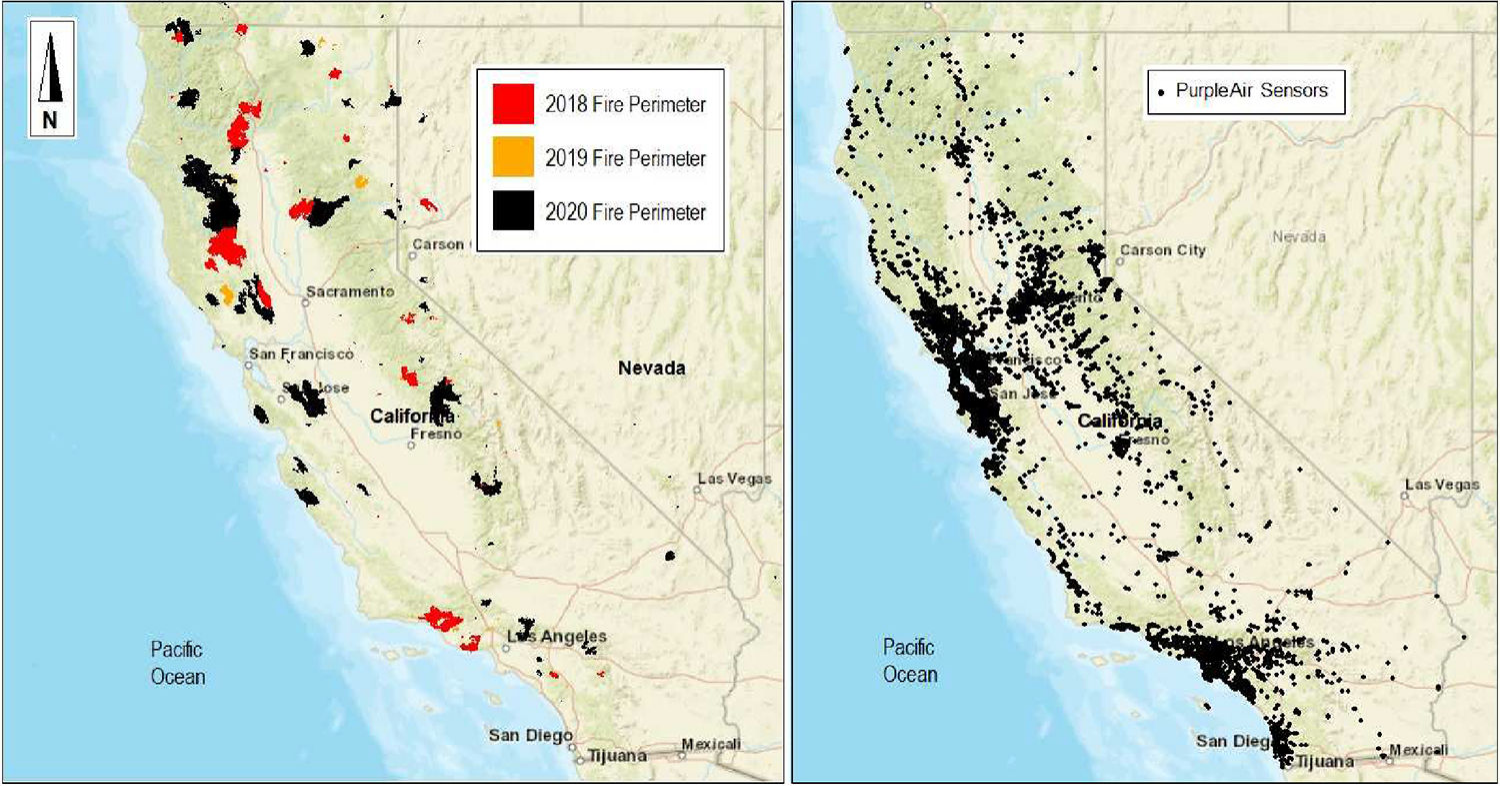
Wildfire burn areas and the distribution of unique PurpleAir sensors for the years 2018–2020 across California.

We examined area burned by wildfires across the entire state of California, USA, over a period of three years (2018–2020) using wildfire perimeter data compiled from the California Fire and Resource Assessment Program (FRAP) and National Interagency Fire Center (NIFC). These agencies estimate the area burned by wildfires according to different methods and protocols, which can result in many non-overlapping burned areas. For instance, FRAP consolidates fire perimeters from multiple agencies including CAL FIRE, the U.S. Forest Service, Bureau of Land Management, and National Park Service based on wildfire perimeter availability and size requirements and is updated once annually. NIFC pools wildfire data similarly, but also makes use of the U.S. Department of Agriculture Forest Service’s National Infrared Operations (NIROPS) program, which determines fire perimeters primarily using thermal infrared imaging from nighttime airborne flights [54]. These perimeters are created based on the need to assist fire management efforts, and are calculated using different methods (e.g., aircrafts, global positioning system handheld devices and/or manual digitizing, post-processing), depending on the availability of resources. In order to obtain complete data relating to the area burned by wildfires across California and to avoid having missed any reported wildfires, we pooled all wildfire records together across both FRAP and NIFC sources. To avoid double counting, all duplicate wildfires were removed during data processing. Wildfire burn area and unique PurpleAir sensor locations for the years 2018–2020 are presented in Figure 1.

### 2.5. Regression Analysis

To understand the individual statistical relationships between census-tract-level socioeconomic characteristics and our outcome variable (CR days/year), univariate regression analysis was performed separately for each variable of interest, the effect estimates and *p*-values of which are presented in the results section. This analysis enabled a description of the spatial pattern of CR exposure as it relates to socioeconomic factors irrespective of causation, collinearity and/or confounding, which is important to understand given the known intercorrelation of such factors and the long history and extensive body of literature that has shown low-income communities and communities of color to be disproportionately exposed to environmental hazards in California and across the United States [8–14]. Following univariate analysis, multivariate regression was performed in order to account for the influence of multiple predictors, their statistical significance and the extent of their relative association.

To address high collinearity between predictors prior to multivariate regression, and therefore minimize the standard errors of effect estimates, a correlation matrix was first generated between all covariates. Where two or more variables were statistically correlated (*p* < 0.05) and exhibited a high Pearson correlation coefficient (r > 0.7), only one variable was retained for multivariate regression. This process resulted in the elimination of highly correlated variables including the percent of residents earning < $35K per year, the percent of residents earning > $100K per year and median home value, which were all highly correlated with median household income. Additionally, the “households without internet” variable was highly correlated with both “households without a computer” and “poverty rate,” and therefore was eliminated prior to modeling. Following this screening process, multivariate regression analysis was performed with the application of stepwise backward elimination of non-significant terms. A formal collinearity analysis of the regression model was subsequently performed using the “collin” and “vif” options in SAS [55]. This enabled an examination of the condition indices and variable inflation factors (VIFs), with condition index values > 10 indicating that weak dependencies may be starting to form and values > 100 indicating that estimates may contain moderate numerical error [56,57].

The same process as that described above was also applied to conduct a multivariate analysis of average PurpleAir-measured daily PM_2.5_ concentrations regressed against wildfire-related predictors. Predictors included the number of wildfires burning within a 100 km radius of a daily PM_2.5_ sensor, the distance of the nearest wildfire to a PM_2.5_ sensor, the area of the nearest wildfire occurring on the same day as a PM_2.5_ measurement, the area of same-day wildfires burning within a 100 km radius of a daily PM_2.5_ sensor, and the total area of all wildfires burning within the state of California on that day. Pre-processing using correlation matrix analysis resulted in the exclusion of “the number of wildfires burning within a 100 km radius,” which was highly correlated with multiple covariates. Following regression modeling, stepwise backward elimination resulted in the elimination of terms that were not statistically significant (*p* < 0.05).

### 2.6. Census Tract Analysis

In order to ultimately identify census tracts facing compounding temperature and air pollution related risk, we first used spatial interpolation to yield spatially continuous surfaces that contained daily PM_2.5_ estimates at the census tract level across the entire state of California for all days within the three-year study period. We calculated the average daily PM_2.5_ concentration across all California census tracts and all days within the three-year study period using the quality-controlled and calibrated PurpleAir data where such measurements were available. The geographic coordinates for the centroid of each census tract were then determined using ArcGIS, yielding 8057 points representing each unique census tract. The coordinates of each point were then assigned to their corresponding calculated daily averages across all days. Using the KRIGE2D procedure in SAS [55] and an input dataset consisting of all census tracts with a known (i.e., measurement-based) average daily PM_2.5_ for a given day, the average daily PM_2.5_ concentration across unknown (i.e., missing) census tracts was then estimated using krig-based spatial interpolation. This was repeated for all 365 days per year for all three years, resulting in a data set consisting of approximately 8.8 million daily PM_2.5_ observations. PM_2.5_ estimates were retained only where daily measurements were missing, while sensor-based averages were retained where PurpleAir measurements existed. Since PurpleAir sensors were available in only a minority of census tracts across the state, approximately 15% of the final daily PM_2.5_ concentration data set consisted of measured data, compared to 85% predicted.

Summary statistics of socioeconomic characteristics were also calculated for each census tract. We focused on the census-tract-level socioeconomic characteristics that could reasonably be expected to render a community at increased vulnerability to heat stress, air pollution, and wildfire. These attributes included residents who identified as Hispanic, Asian, African American, or Native American, residents who reported speaking no or limited English, residents who did not have health insurance coverage, residents under five years of age and those over sixty-five years of age, renter-occupied housing units, residents with a college education or higher, as well as whether or not households had computers and internet. We also calculated statistics relating to economic factors such as unemployment rate, poverty rate, median household income, and median home value.

In order to identify days with compounding temperature and air pollution risks (hence-forth, “compound risk” days), we first calculated summary statistics for socioeconomic factors across census tracts that were grouped according to the number of days that they experienced both an average daily concentration of PM_2.5_ > 35 μg/m^3^ and a maximum daily temperature ≥ 35 °C (95 °F) over the three-year study period. For temperature, the chosen temperature cutoff to represent “heat days” is consistent with prior work that used a 35 °C (95 °F) temperature cutoff [2,58]. For PM_2.5_, our cutoff corresponds with the EPA’s 24-hour PM_2.5_ standard [59]. Socioeconomic factors were then compared across census tracts whose number of CR days fell within discrete ranges (e.g., 1–5 days, 6–10 days, etc.) using scatter plots with accompanying trendlines. The spatial distribution of census tracts that were among the highest risk when considering these compound factors was compared to the spatial distribution of the “high-impact” census tracts identified through our previous work (which examined direct wildfire burn area) in order to examine the potential geographic overlap between these compound health threats.

## 3. Results

**Figure 2.**
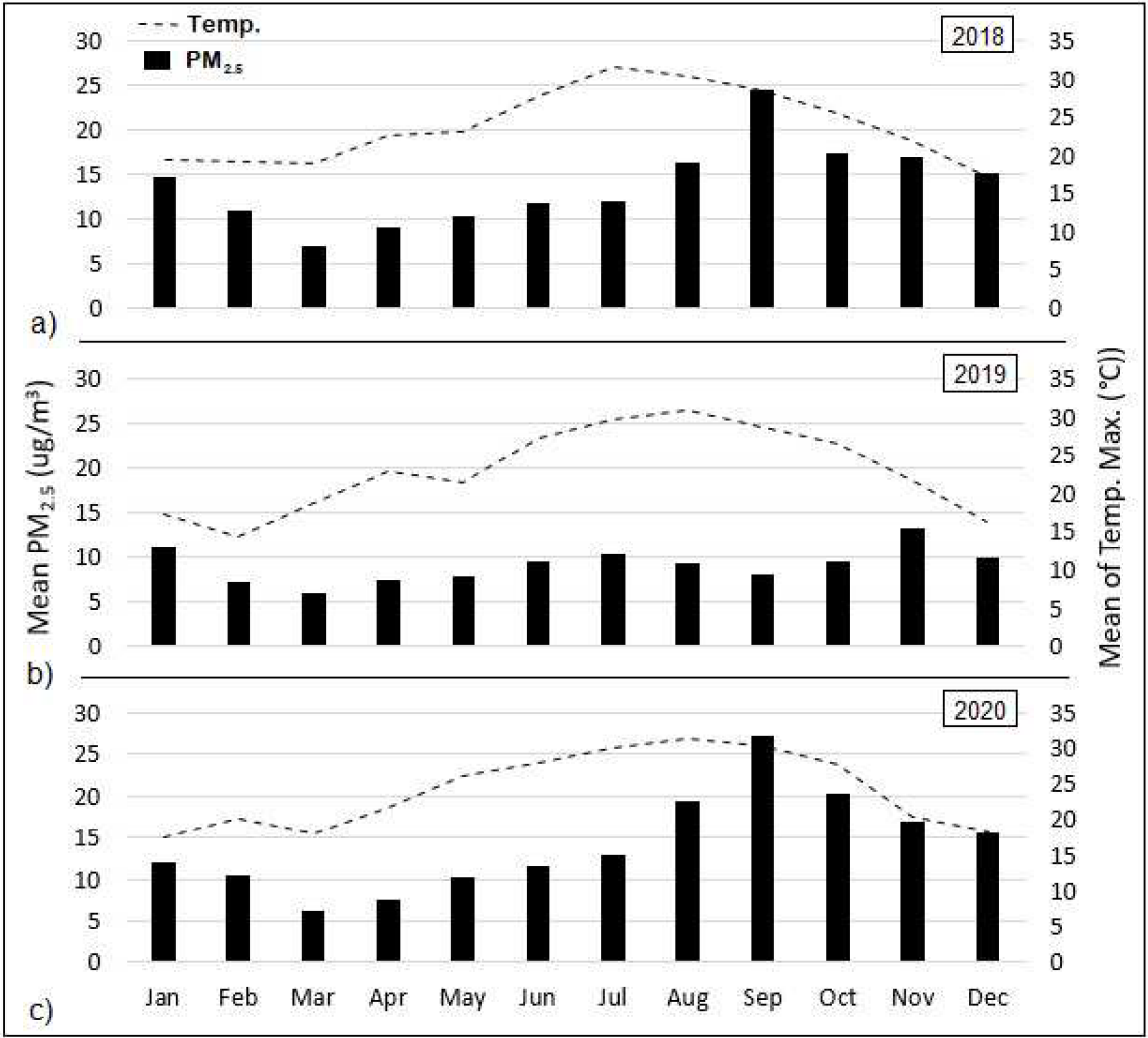
Monthly average PM_2.5_ concentrations across entire state of California by year, based on aggregation of sensor measurements and spatial interpolation.

Figure 2 presents monthly average PM_2.5_ concentrations and surface temperature across the entire state of California by year using an aggregation of both sensor-based measurements and interpolation. As shown, the late summer and early fall months tended to reflect the highest PM_2.5_ concentrations whereas spring demonstrated the lowest levels. In contrast, monthly temperatures peaked during the summer months of July and August. Compared to 2019, the years of 2018 and 2020 exhibited far higher PM_2.5_ concentrations, with annual mean concentrations (14.0 ± 0.2 μg/m^3^) that were approximately 55% higher than that of 2019 (9.1 μg/m^3^), coinciding with the pattern of annual wildfire activity throughout the state (2018 and 2020 being record-breaking wildfire years, and 2019 relatively inactive) [20]. When evaluating the monthly 95th percentile PM_2.5_ concentrations (statewide averages), we similarly saw much higher peaks in 2018 and 2020 (65.0 to 81.9 μg/m^3^ in September) relative to 2019 (25.3 μg/m^3^). Monthly mean PM_2.5_ levels showed similar lows of 6.5 ± 0.5 μg/m^3^ (occurring in March) across all three calendar years, whereas the highest monthly average of 25.8 ± 1.4 μg/m^3^ occurring in September for years 2018 and 2020 was roughly two-times higher than the highest monthly mean of 13.3 μg/m^3^ that occurred in 2019 (November). For the two years with the highest air pollution, PM_2.5_ concentrations were greatest during the months of August, September, and October, coinciding with the peak wildfire season. When examining the temperature pattern, neither the annual averages nor the seasonal extremes appeared visibly different from year to year.

**Figure 3.**
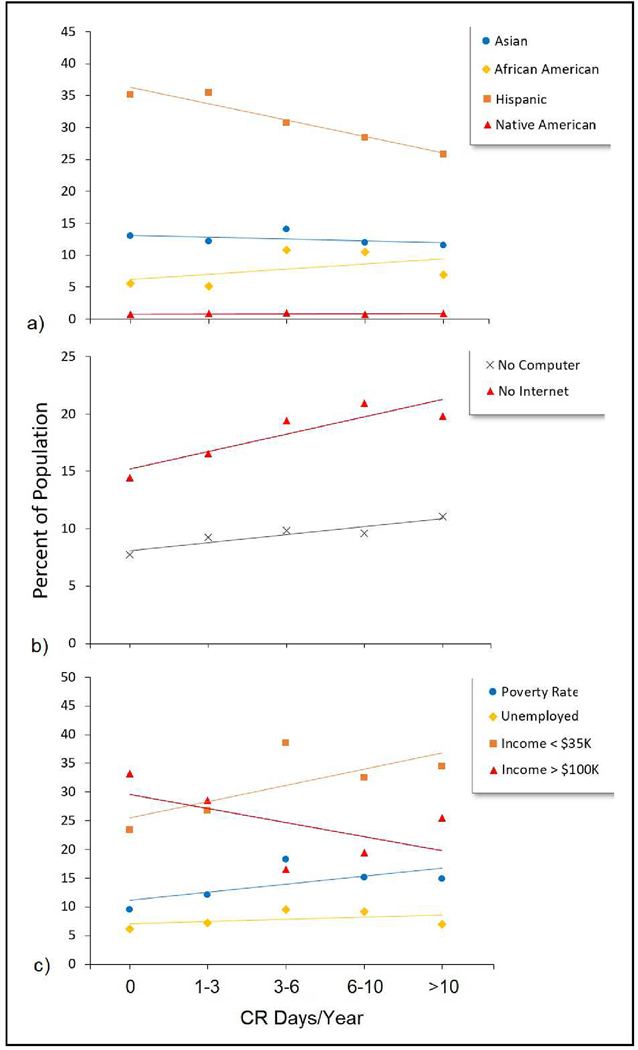
Percent of (**a**) minority populations, (**b**) temperature and air pollution vulnerability indicators and (**c**) economic indicators averaged across census tracts grouped by frequency of compound-risk (CR) days per year. Trendlines are presented to illustrate the direction of the slope and should not be interpreted as indicating a linear relationship.

Figure 3a presents the percent of minority populations across census tracts grouped according to their annual frequency of CR days from 2018 to 2020. On average, there was a positive correlation with CR days when considering African American residents, of which there was a 76% greater proportion (based on trendline) in census tracts that had the highest frequency of CR days per year (CR days > 10) compared to the lowest (CR days = 0). Though not shown in Figure 3, trendlines were similar for residents who reported speaking no or limited English. There was no trend as it relates to female/male differences.

Figure 3b illustrates increased temperature and air pollution vulnerability among census tracts that experienced the highest compound risk over the 3-year study period as indicated by higher proportions of households without internet and computers among census tracts with a higher frequency of CR days. Specifically, there was a relative increase of 37% and 43% in the proportion of households without internet and without computers, respectively, within census tracts with the greatest frequency of CR days compared to census tracts with the lowest frequency of CR days (based on trendline). Although not shown, other vulnerability indicators such as the proportions of child residents (age < 5 years), renter-occupied housing units, and residents without a college education exhibited a similar pattern of increase, albeit more modest (~10%), when comparing opposite ends of the CR frequency trendline. The proportion of elderly (age > 65 years) residents exhibited no discernable pattern when examining the CR trendline.

**Figure 4.**
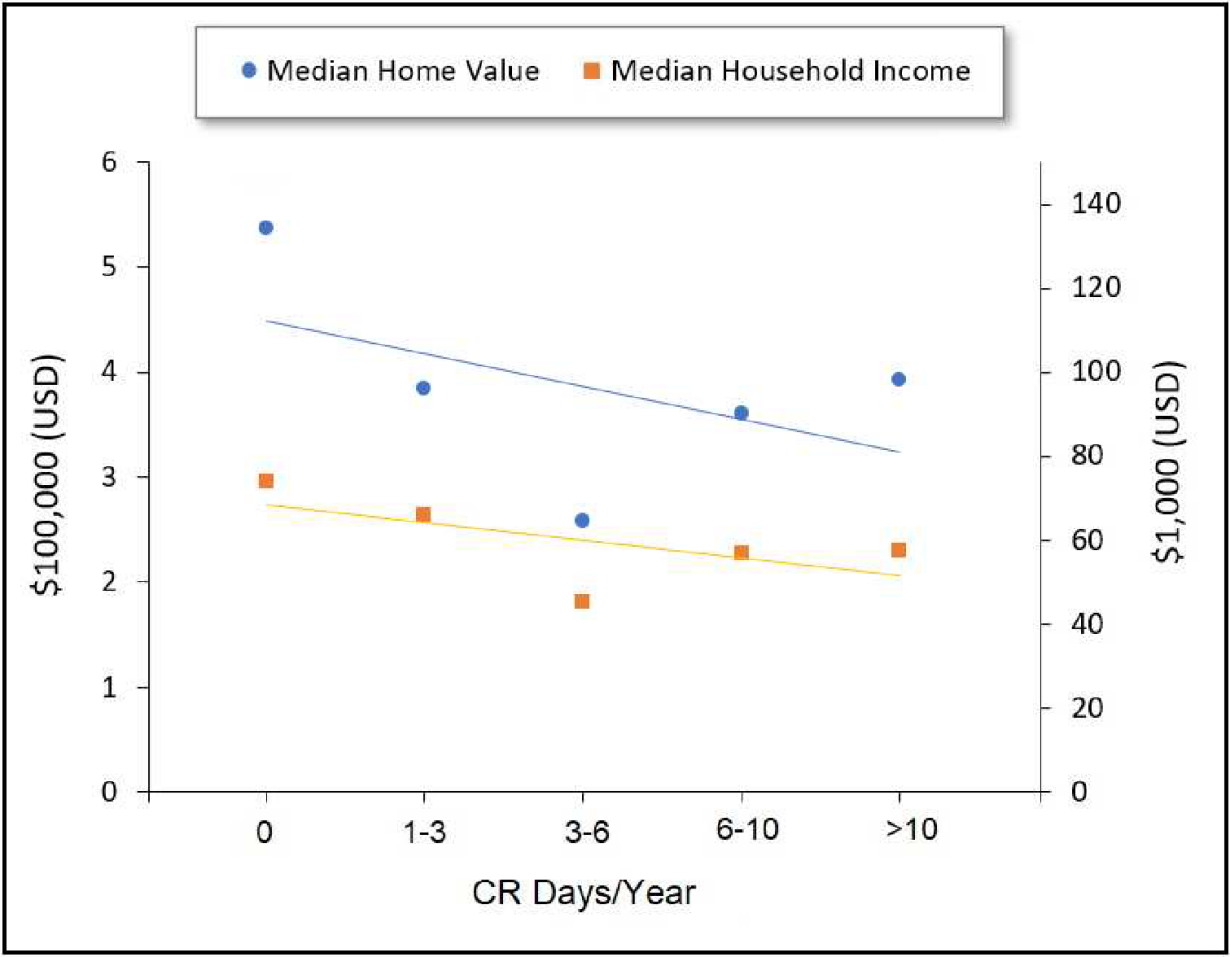
Percent of economic indicators averaged across census tracts grouped by frequency of compound-risk (CR) days per year. Trendlines are presented to illustrate the direction of the slope and should not be interpreted as indicating a linear relationship.

Greater vulnerability among census tracts with a higher annual frequency of CR days was also apparent for the economic characteristics presented in Figures 3c and 4, as reflected by higher proportions of low-income residents, unemployed residents, and residents living in poverty, lower proportions of high-income residents, lower median household incomes, and lower home values on average. When considering the slope of each trendline, the most dramatic slopes were those relating to income, for which there was a 44% increase and 28% decrease on average in the proportion of low-income and high-income households, respectively, within census tracts at the opposite ends of the CR day frequency groupings. When considering poverty rate, while the absolute percent change across CR categories was less dramatic, the magnitude of the change when comparing census tracts at opposite ends of the CR frequency grouping was dramatic, representing a 56% increase in the poverty rate among census tracts with the highest frequency of CR days.

Figure 4 presents the percent of other economic indicators averaged across census tracts grouped by annual CR frequency. As shown, median home value decreased by $34K for each increase in CR category on average, corresponding to a roughly $160K decrease from the lowest to the highest CR category, while median household income decreased by roughly $5K for each one-category increase in compound risk on average, corresponding to an $18K decrease from the lowest to the highest CR category. Summary data on which Figures 3 and 4 are based is available in Table S1 of the supplemental materials section.

Following grouped correlation analysis, Table 1 presents results of effect estimates and statistical significance following ungrouped (all point observations included) univariate regression analysis for the socioeconomic variables presented in Figures 3 and 4. Results show statistical significance (*p* < 0.05) for all variables except for two race/ethnicity terms (% African American residents and % Hispanic residents). Economic variables (e.g., poverty pate, percent unemployed, etc.) tended to show the greatest magnitude of effect with CR days compared to other variables. Importantly, since the results presented in Table 1 were derived from univariate analyses, they do not control for multiple variables that may exhibit collinearity.

Table 2 presents a subset of variables that were determined to exhibit minimal collinearity and subsequently shown to have a statistically significant association with the outcome variable (CR days/year) following multivariate regression analysis. As shown, after accounting for the influence of multiple variables, the race/ethnicity terms no longer remained positively associated with CR days, while poverty rate and unemployment rate remained positively associated with CR days, as did the percent of households without a computer and the percent of residents under the age of five. A comparison of the effect estimates in Table 2 demonstrates the economic terms (i.e., unemployment and poverty) to be the most positively associate with CR days.

**Figure 5.**
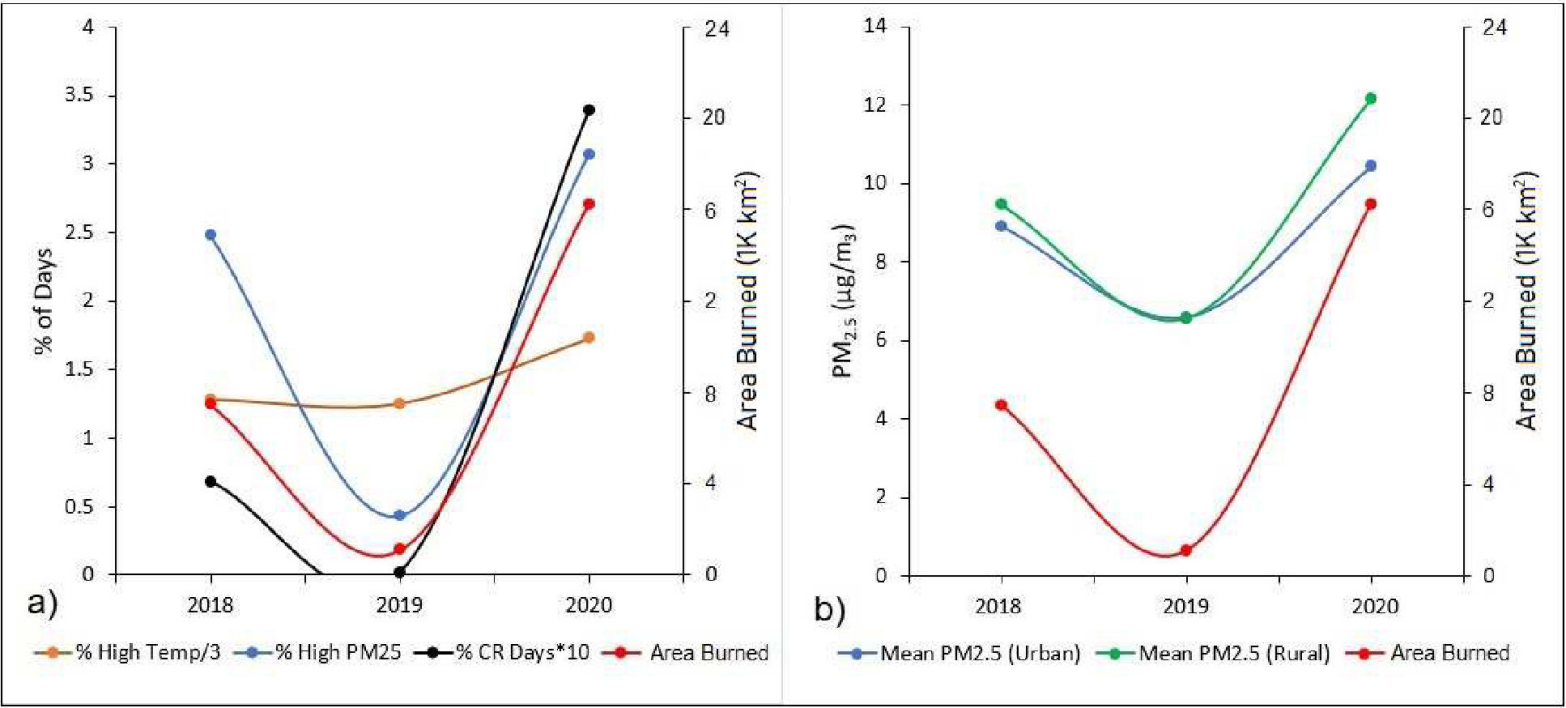
Scatter plots of total wildfire burn area across California compared to the percent of (**a**) high temperature days (max daily temp. > 35 °C), high PM_2.5_ days (mean daily PM_2.5_ > 50 μg/m^3^), and compound risk (CR) days (both high temp. and high PM_2.5_) within all California census tracts and (**b**) mean annual PM_2.5_ concentrations within both urban and rural census tracts in California from 2018 to 2020.

Figure 5 presents scatter plots comparing total wildfire burn area across California with the percent of high temperature days, high PM_2.5_ days, and CR days on an annual basis across the state. As shown, the annual change in all four variables exhibited similar patterns throughout the study period—namely, a dip in 2019 followed by a dramatic increase in 2020. These patterns were most similar when comparing PM_2.5_ with burn area and CR days, whereas the changes were less pronounced when comparing these variables with temperature.

Figure 5b allows for a similar comparison with total wildfire burn area, except in this case juxtaposing annual average PM_2.5_ concentrations (using only PurpleAir measurement data) broken down by urban and rural census tract designations. As demonstrated, annual state-averaged PM_2.5_ concentrations were higher in 2018 and 2020 across rural census tracts compared to urban census tracts, whereas PM_2.5_ concentrations were approximately the same across both regions during the relatively quiet wildfire year of 2019. While annual average PM_2.5_ for rural and urban regions both exhibited sharp spikes in 2020, the more pronounced increase in PM_2.5_ across rural areas more closely mirrored the annual change (slope of trendline) in total wildfire burn area across the state than did that of urban areas. When comparing census tract-level daily PM_2.5_ concentrations with the U.S. EPA’s 24-hour PM_2.5_ standard, the percent of daily exceedances (PM_2.5_ > 35 μg/m^3^) corresponded to 3.6%, 0.7% and 4.9% for 2018, 2019, and 2020, respectively, compared to 3.1% over all years.

**Figure 6.**
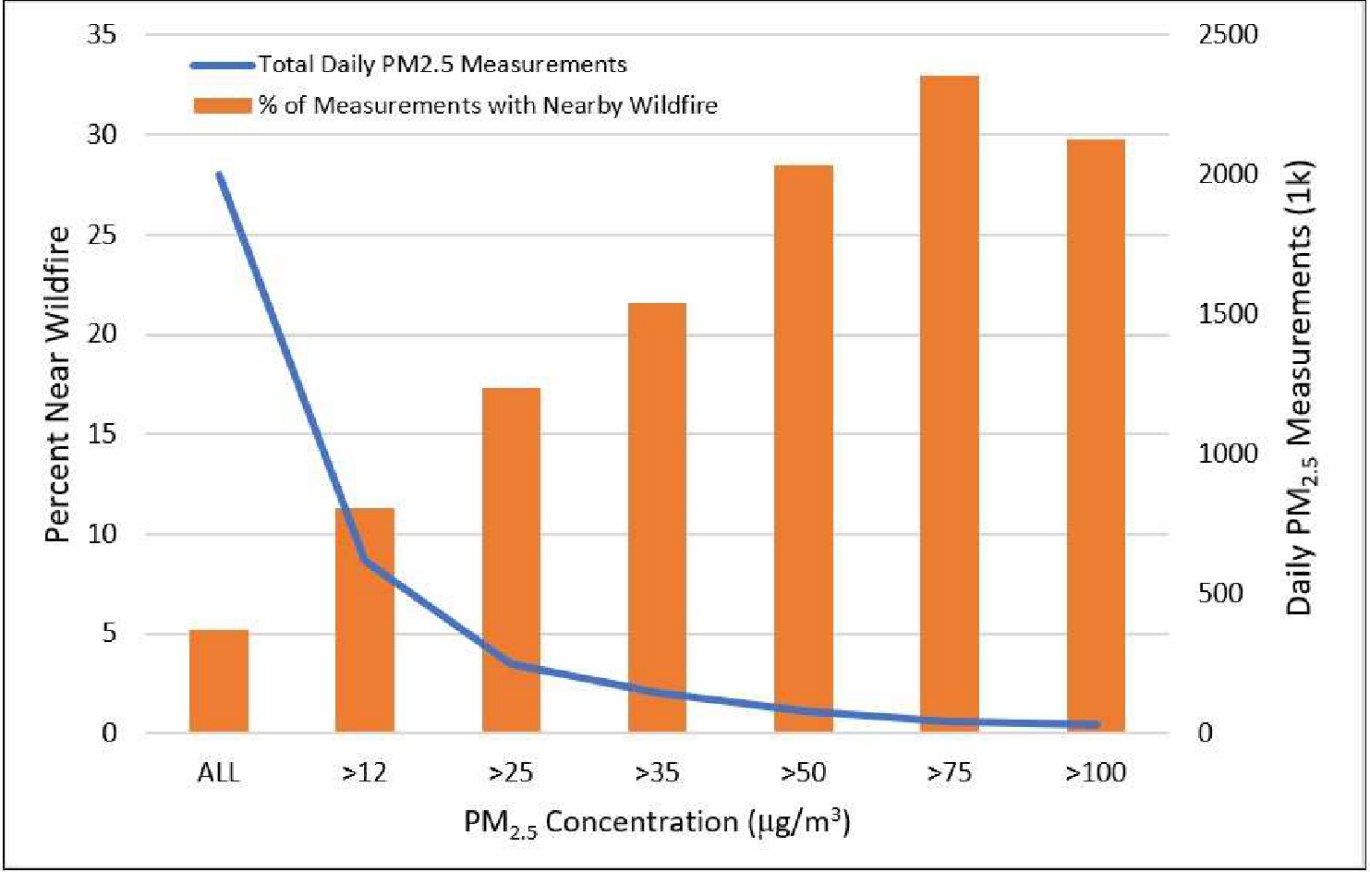
Number of sensor-based PM_2.5_ observation days where average daily measurements exceeded various concentration thresholds (blue line) and the percent of those measurements (orange bars) where a wildfire was reported within a 100 km radius.

Figure 6 depicts the number of PM_2.5_ observations days where average daily PM_2.5_ measurements exceeded various concentration thresholds (blue line) and the percent of those measurements (orange bars, termed “Percent Near Wildfire) where a wildfire was reported within a 100 km radius. Moving from left to right, each successive bar of the histogram depicts a percentage relating to a shrinking dataset (as data becomes increasingly restricted after considering only measurements >12 μg/m^3^, >25 μg/m^3^, etc.). As shown, only approximately 5% of total (~2000K) PM_2.5_ measurements were reported to have a wildfire within 100 km. However, when we examined the roughly 500K PM_2.5_ measurements above 12 μg/m^3^, this percent more than doubled (11.3%). The percent of measurement days characterized by a nearby wildfire continued to increase markedly as we restricted to more heavily polluted measurement days. When considering only PM_2.5_ measurements greater than 75 μg/m^3^, one-third of such measurements had a wildfire reported within 100 km. Although not shown, an examination of CR days across census tracts where PA sensors existed similarly showed a high proportion (64%) of CR days to fall on days in which a wildfire was reported within 100 km.

**Figure 7.**
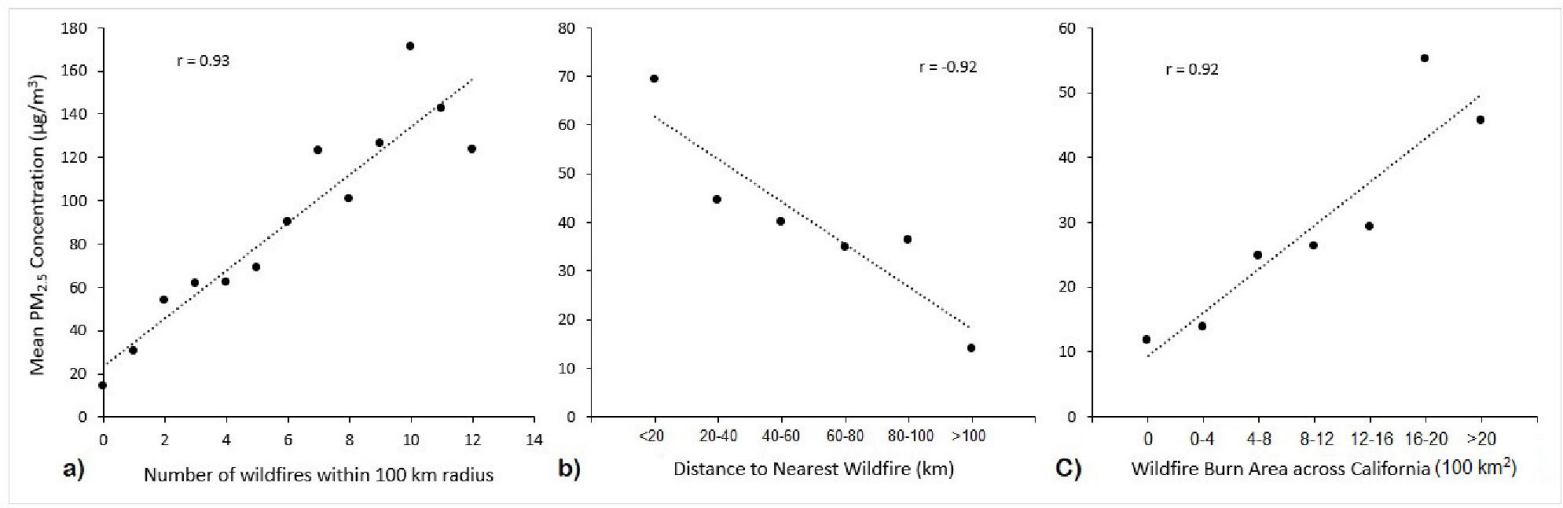
Scatter plots of average daily PM_2.5_ concentrations as measured by PurpleAir sensors grouped according to the (**a**) number of wildfires reported within 100 km of a sensor on the same day of PM_2.5_ measurement, (**b**) the distance of a sensor to the nearest wildfire on the same day of PM_2.5_ measurement, and (**c**) the cumulative area of wildfires that burned across California during the same week of measurement.

Figure 7 presents select scatter plots of average PM_2.5_ concentrations as measured by PurpleAir sensors grouped according to discrete graduations relating to three different wildfire metrics. Figure 7a presents daily PM_2.5_ concentrations averaged across PurpleAir sensors that were divided into 14 groups based on the number of wildfires reported within 100 km of each sensor on a given PM_2.5_ measurement day. As shown, the plot demonstrates a positive correlation with a high Pearson correlation coefficient (r = 0.93). Figure 7b presents a very similar plot, in this case with PurpleAir sensors grouped based on the distance of each sensor to the nearest reported wildfire (within a 100 km buffer). Similarly, a strong correlation is reported (r = −0.92), albeit in the opposite direction given the different x-axis, which is consistent with the influence shown in Figure 7a. Figure 7a,b enable an examination of the correlation between PM_2.5_ pollution and local wildfire impacts, whereas Figure 7c enables an assessment of such pollution and its correlation with impacts that may be either short- or long-range. In Figure 7c, PM_2.5_ concentrations were averaged across PurpleAir sensors that were grouped based on the cumulative area of wildfires that burned across the entire state of California during the same week that each sensor measurement was collected. As shown, the plot demonstrates a positive correlation with a high Pearson correlation coefficient (r = 0.92). Figure S2 of the supplemental materials section provides graphics similar to Figure 7c, yet depicting only local impacts (total burn area of the nearest wildfire and total burn area within a 100 km radius), which showed a pattern very similar to that of Figure 7c, albeit with greater PM_2.5_ concentrations per unit of burned area.

Table 3 presents the effect estimates (EE) and *p*-values following multivariate analysis of average PurpleAir measurements regressed against various wildfire-related predictors. Results showed the distance to the nearest wildfire, the area of the nearest wildfire and the area of all wildfires in California to be significantly (*p* < 0.05) associated with daily PM_2.5_ concentrations. Of note, the total area of wildfires across California exhibited a higher effect estimate than the area of the nearest wildfire. Collinearity analysis demonstrated only weak collinearity (highest condition index = 13) between predictors.

**Figure 8.**
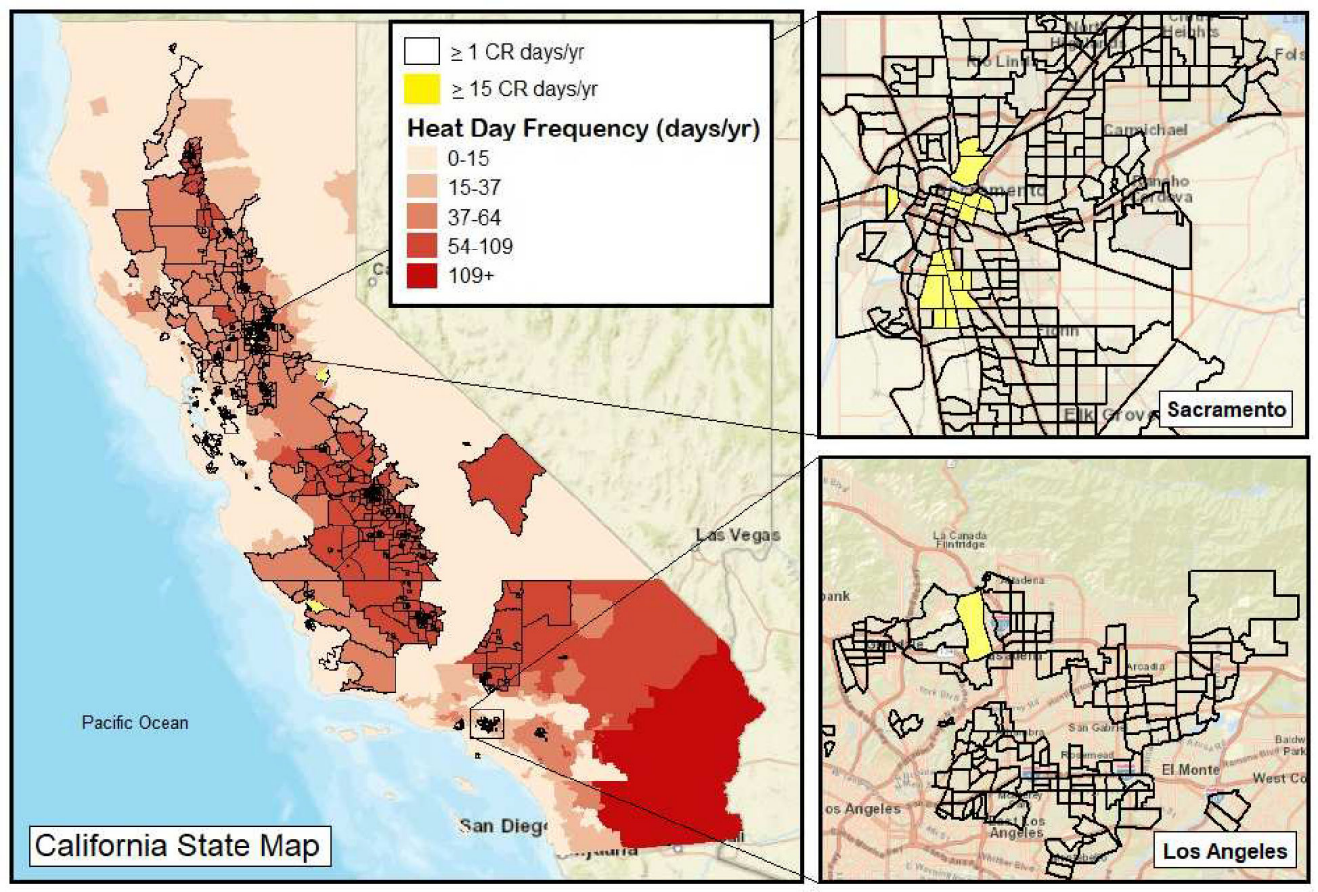
Compound risk days overlaid with heat days at the census tract level.

Figure 8 presents a map of California in which the annual frequency of “heat days” appears in varying shades of red, while the census tracts that experienced ≥ 1 and ≥ 15 CR day(s) per year are depicted in black outlines and yellow highlights, respectively. As shown, the highest frequency of CR days tended to be those with small perimeters which are located in highly urbanized environments such as Los Angeles and Sacramento. What is more, the CR frequency was generally highest across inland areas that were distant from coastal and mountainous regions.

Although urban census tracts tend to be small, census tract boundaries tend to encompass similarly sized populations. In the present case, the average census tract population across California during this study period was approximately 4900 people, with a standard deviation of 2200 people. When considering census tracts with ≥ 1 CR days per year, the total exposed population across the state equaled 1.8 million people, whereas the population within census tracts that experienced ≥ 15 CR days per year amounted to approximately 61,000 people.

## 4. Discussion

### 4.1. Census Tract Analysis

Our univariate assessment of socioeconomic factors demonstrated that census tracts incurring a higher frequency of compound risk days, as indicated by elevated temperature and PM_2.5_ concentrations, tended to have statistically significantly higher proportions of Native American residents and lower proportions of Asian residents. As it relates to Native American residents, this pattern may be due to their tendency to reside in more rural regions of the state, which see greater temperature and wildfire/smoke [60].

Additional findings from univariate analysis showed that census tracts with a higher frequency of CR days had higher proportions of low-income residents, lower proportions of high-income residents, as well as lower median household incomes and home values. Similarly, an assessment of vulnerability-related characteristics demonstrated higher proportions of child residents (age < 5) and residents/households without computers, internet, and a college education, along with lower proportions of renter-occupied housing units and higher rates of poverty and unemployment among census tracts experiencing the highest frequencies of CR days. The proportion of elderly residents exhibited no association with CR days.

After addressing potential collinearity, results from multivariate analysis enabled an assessment of the relative influence of multiple variables, showing economic factors (e.g., poverty rate and unemployment) to be most positively associated with the frequency of CR days followed by the proportion of child residents and the proportion of households without computers, whereas race/ethnicity was not positively associated with CR days.

Collectively, these findings are important as they underscore the potential for disproportionate health impacts relating to the compounding effects of heat stress and air pollution exposure among vulnerable and socioeconomically disadvantaged communities, in turn highlighting issues of environmental justice that must be considered given the anticipated future increase in human-induced global warming and related air pollution. For instance, households without computers (and internet) may be less likely to be aware of, and therefore prepare for, an approaching heat-wave and/or wildfire or smoke plume, as prior research has shown online social media platforms to be useful tools of rapid on-the-ground citizen reporting and dissemination of disaster information (in some cases publishing news before traditional media channels [61]. What is more, low-income households may be less able to run air conditioning during high-temperature days due to the cost of electricity or to purchase air purifiers during a wildfire or other major air pollution event due to the cost of such products. In some low-income communities, including tribal lands, antiquated and/or unreliable electric infrastructure leads to power outages during heatwaves which can also leave residents vulnerable, particularly those living in mobile homes which are often poorly insulated.

Lastly, the higher proportion of children reported among census tracts with a high frequency of CR days is a public health concern given the increased sensitivity of children (particularly infants and toddlers) to heat stress, along with their higher inhalation rate (relative to body size) which make children disproportionately vulnerable to the adverse impacts of air pollution exposure [62].

The prevalence of CR days in census tracts located in urban environments suggests that non-wildfire-related emissions sources are likely of great importance to the frequency of compound risk days in many areas. Having said that, a statewide analysis of the frequency of high PM_2.5_ days across all census tracts demonstrated an annual pattern that closely tracked that of total wildfire burn area across the state, suggesting that wildfires are nonetheless a likely contributor to poor air quality and compound risk days in California. This finding was reinforced by results of annual PM_2.5_ concentrations averaged across PurpleAir measurements (rather than interpolated data), which showed higher PM_2.5_ levels in rural as opposed to urban census tracts on average. Rural census tracts are also where wildfires tend to occur more frequently [20]. In general, the change in PM_2.5_ (slopes of trendlines) tended to mirror that of wildfire activity more closely when considering rural compared to urban census tracts, suggesting a potentially greater role of wildfire activity as a contributor to rural PM_2.5_ concentrations.

Importantly, results from our analysis show that a high fraction of days with elevated PM_2.5_ occurred when a wildfire was burning nearby (within a 100 km radius). This was particularly noticeable when restricting data to only days with 75 μg/m^3^ or higher PM_2.5_ averages, for which one-third of measurements were characterized as having a nearby wildfire, which is over six-times higher compared to the proportion across all measurement days. When examining sensor-measured PM_2.5_ concentrations according to their number of same-day reported nearby wildfires, the distance of each sensor to the nearest wildfire, as well as the cumulative area of same-week wildfires that burned across the entire state, strong positive correlations were observed. What is more, an examination of CR days over our study period found that the majority occurred on days in which a nearby wildfire was reported. Among wildfire-related covariates, multivariate regression analysis identified the distance to the nearest wildfire and the area of both the nearest wildfire and total wildfire throughout California to be predictive of daily sensor-based PM_2.5_ concentrations.

Collectively, these findings suggests that PM_2.5_ pollution and the occurrence of CR days in California were strongly influenced by wildfire events during the study period. Reinforcing this conclusion was our finding from our assessment of interpolated censustract level PM_2.5_ concentrations relative to the EPA’s 24-hour ambient PM_2.5_ standard, which found much higher percentages of daily exceedances during the relatively active wildfire years of 2018 and 2020 compared to 2019. As shown in our prior work, 2018 and 2020 were record-breaking wildfire years whereas 2019 was a relatively inactive year [20]. Overall, these findings are consistent with results from prior studies that have shown wildfire events to explain the majority of PM_2.5_ on days that exceed regulatory standards, as well as studies in both California and nationally which have shown wildfire-related air pollution to be detracting from what would otherwise be reductions in long-term ambient air pollution trends in fire-prone regions [63–66].

When examining CR days, the relative increases in 2018 and 2020 appeared to be driven mostly by changes in the frequency of high PM_2.5_ days rather than high temperature days. That is, the annual change in PM_2.5_ concentrations, wildfire activity, and CR days all exhibited similar patterns throughout the study period—namely, a sharp dip in 2019 followed by a dramatic increase in 2020. For temperature, however, the change was much more subtle over the study period. Collectively, these findings underscore air pollution, either related to or independent of wildfire activity, as the main determinant of the frequency of compound risk. This suggests that tracking and reducing air pollution may be a reliable way of predicting and avoiding, respectively, the occurrence of CR days across California. What is more, while reducing ambient temperature (e.g., increasing greenspace) may represent a costlier and more logistically challenging way to avoid CR days, the risk related to CR days can nonetheless be reduced by household adaptive measures such as staying indoors and/or using indoor air conditioning on CR days. Having said that, extensive research has shown that practical urban planning strategies such as the planting of vegetation to increase greenspace can be highly effective in combatting high temperatures and minimizing the urban heat island effect [67–69]. As it relates to regional differences, communities inland from coastal and mountainous areas showed the highest frequency of CR days, underscoring the importance that such communities and their elected officials should place on adaptive measures to protect public health.

### 4.2. Strengths and Limitations

A strength of this analysis is the use of high-resolution daily PM_2.5_ and temperature data, along with daily wildfire data, which enabled the estimation of high-risk census tracts across the entire state of California. Another strength is the assessment of socioeconomic correlations at a high spatial resolution (census tracts level), which allowed for the identification of communities potentially facing health inequities. Lastly, assessing the frequency of the spatiotemporal overlap between high air pollution and high temperature days offered an evaluation of the potential compounding risks that may render communities more vulnerable to hospitalization and disease than if either threat was considered separately.

One limitation of this study is the estimation, as opposed to measurement, of a high number of census-tract-level PM_2.5_ observations. This may have resulted in the misclassification of high- or low-risk census tracts across certain areas and/or days where spatial interpolation was conducted at a far distance and/or based on a low number of surrounding measurements. Since a high fraction of PM_2.5_ sensors were located in urban areas, urban/rural differences may have also contributed to potential exposure misclassification and may limit the representativeness of our study for rural populations. Additionally, this study only evaluated the potential (i.e., exposure) for health impacts, as opposed to examining actual health outcomes. Future work should be dedicated to examining whether the census tracts with the highest frequency of CR days are in fact reporting greater hospital admissions, morbidity, and mortality. Another limitation is the potential for temporal mismatch given that a single 5-year averaged ACS dataset was used to represent the entire study period. Moreover, PurpleAir sensors were disproportionately present in urban areas, which may have introduced bias as well as resulted in the missing of some wildfire-related high-PM episodes (which more often occur in rural areas where fewer air sensors exist) [70].

Regarding temperature, it is also important to note that our use of >35 °C as a high-temperature threshold does not account for adaptation and sensitivity of different sub-populations, which may reduce temperature-related risk in certain regions. Additionally, despite the census tract being a high-resolution, temperature estimates at this spatial scale cannot capture the heat-island effect, which may have underestimated temperature-related risk in urban areas. Lastly, an equal weight was assigned to temperature and air pollution in this analysis in order to estimate CR values. However, this assumption may not reflect the true health risk borne by communities with differing health outcomes and risk factors.

## 5. Conclusion

We used low-cost air monitoring sensors and meteorological data to help identify the census tracts in California that most frequently experienced the compounding impacts of high temperature and PM_2.5_ pollution on a daily basis from 2018 to 2020. Results showed socioeconomically disadvantaged census tracts to incur a higher frequency of CR days compared to other groups, with multivariate regression analysis demonstrating economic factors (e.g., poverty and unemployment) to be most positively associated with the frequency of CR days followed by the proportion of child residents and the proportion of households without computers. The frequency of compound-risk days and elevated daily PM_2.5_ concentrations appeared to be predominantly related to the occurrence of nearby wildfires. Findings from this study are important to policymakers and government agencies who preside over the allocation of state resources as well as non-governmental organizations, public health officials and community groups seeking to empower residents and establish climate resilient communities.

## Supplementary Material

Supplemetnal materials

## Figures and Tables

**Table 1.** Effect estimates and *p*-values for socioeconomic variables following univariate regression with outcome variable (CR days/year).

	Effect Estimate	*p*-Value

Asian Residents (%)	−0.00474	0.0011
African American Residents (%)	0.00188	0.4584
Hispanic Residents (%)	0.00086	0.3099
Native American Residents (%)	0.02302	0.0147
Households without Computer (%)	0.03320	<0.0001
Households without Internet (%)	0.02240	<0.0001
Poverty Rate (%)	0.03108	<0.0001
Unemployed (%)	0.05774	<0.0001
Income < $35K (%)	0.02134	<0.0001
Income > $100K (%)	−0.01508	<0.0001
Median Home Value ($100K)	−0.13890	<0.0001
Median Household Income ($10K)	−0.07261	<0.0001
Residents without College Degree (%)	0.01157	<0.0001
Residents < Age 5 (%)	0.00067	<0.0001
Residents > Age 65 (%)	−0.000091	0.1094

**Table 2.** Effect estimates and *p*-values for multivariate socioeconomic model after stepwise backward elimination of non-significant terms.

	Effect Estimate	*p*-Value

Intercept	0.45298	<0.0001
Hispanic Residents (%)	−0.01257	<0.0001
African American Residents (%)	−0.01265	<0.0001
Households without a Computer (%)	0.02272	<0.0005
Unemployment Rate (%)	0.03616	<0.0001
Poverty Rate (%)	0.03265	<0.0001
Residents < Age 5 (%)	0.00069	<0.0001

**Table 3.** Effect estimates (EE) and *p*-values following multivariate analysis of wildfire terms.

	EE	*p*-Value

Intercept	49.49	<0.001
Distance to nearest wildfire (10 km)	−1.66	<0.001
Area of nearest wildfire (10 km^2^)	0.14	<0.001
Area of all wildfires in California (10 km^2^)	0.17	<0.001
