# Supplementary material for: Compound Risk of Air Pollution and Heat Days and the Influence of Wildfire by SES across California, 2018–2020: Implications for Environmental Justice in the Context of Climate Change
Source: Climate (Basel). Author manuscript; Available in PMC 2024 Mar 7. (PMC10919222; doi:10.3390/cli10100145)
Supplement: Supplemetnal materials [file NIHMS1967156-supplement-Supplemetnal_materials.pdf]

Supplemental Materials

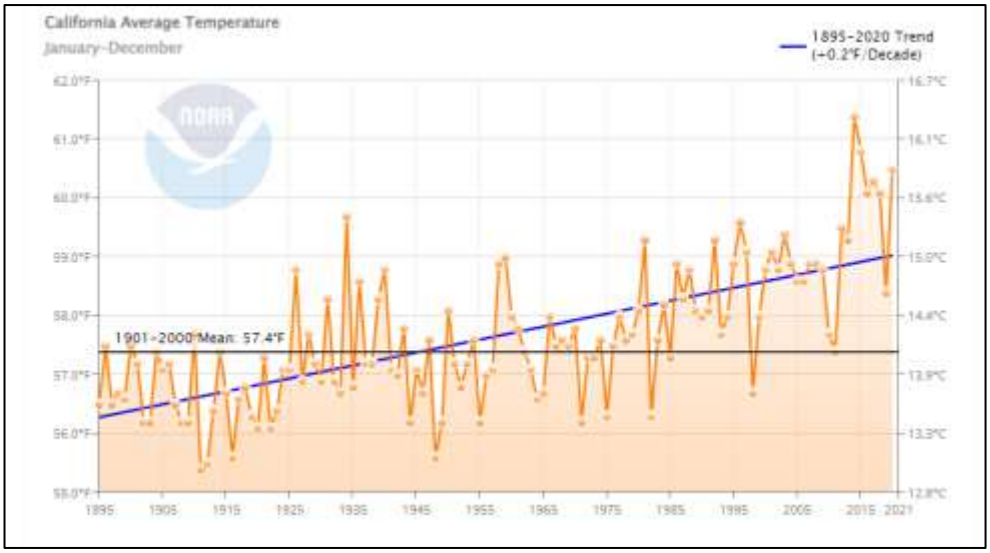

Figure S1. California annual average temperature (Source: U.S. National Oceanic and Atmospheric Administration).

Table S1. Summary of socioeconomic characteristic across census tracts grouped by the frequency of their estimated CR days/year.

	CR Days/Year				
	0	0-1	1-6	6-10	>10
Asian Residents (%)	14.0	13.2	15.2	12.9	12.5
Hispanic Residents (%)	37.9	38.2	33.1	30.6	27.8
Native American Residents (%)	0.8	0.9	1.0	0.8	0.9
African American Residents (%)	6.0	5.6	11.6	11.3	7.5
Households without Computer (%)	8.3	10.0	10.6	10.3	11.9
Households without Internet (%)	15.6	17.9	20.9	22.6	21.4
Poverty Rate (%)	10.2	13.0	19.7	16.2	16.0
Unemployed (%)	6.6	7.7	10.2	9.9	7.5
Income < $35K (%)	25.1	28.8	41.5	35.0	37.1
Income > $100K (%)	35.7	30.7	17.8	21.0	27.4
Median Home Value ($)	79,781	70,920	48,794	61,278	61,898
Median Household Income ($)	579,351	413,275	278,269	387,746	422,588

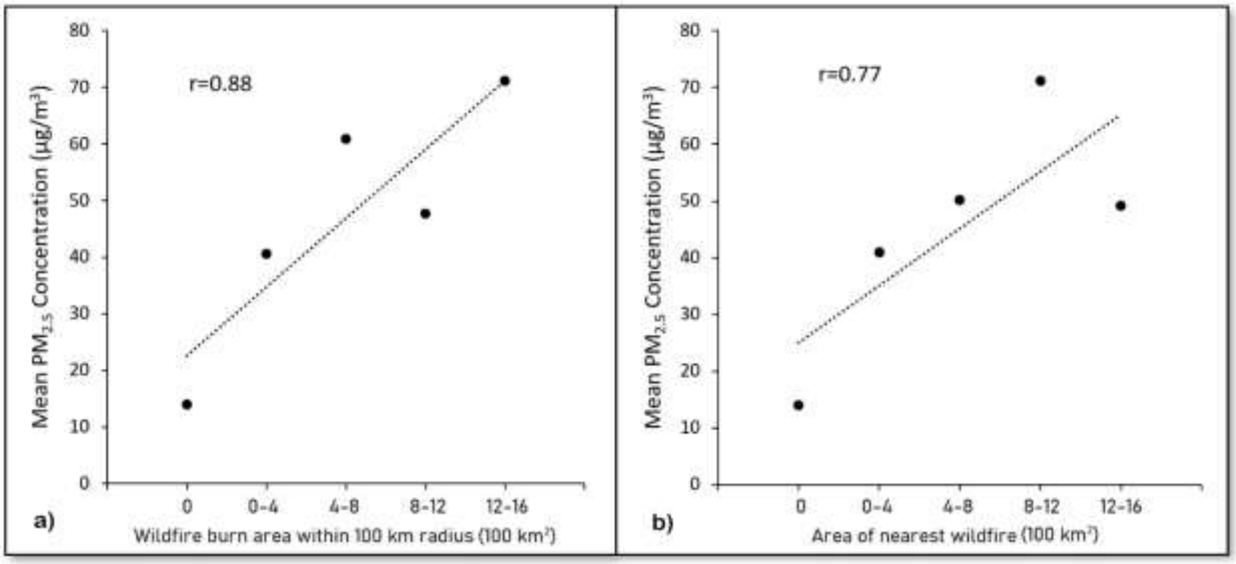

Figure S2. Scatter plots of average daily $PM_{2.5}$ concentrations as measured by PurpleAir sensors grouped according to the a) wildfire burn area reported within 100 km of a sensor on the same day of $PM_{2.5}$ measurement and b) the same-day burn area of the nearest wildfire.
